# Supplementary material for: Diagnostic performance of molecular and serological tests of SARS-CoV-2 on well-characterised specimens from COVID-19 individuals: The EDCTP "PERFECT-study" protocol (RIA2020EF-3000)
Source: PLoS One. 2022 Sep 21;17(9):e0273818. doi: 10.1371/journal.pone.0273818 (PMC9491536; doi:10.1371/journal.pone.0273818)
Supplement: S1 File — (PDF) [file pone.0273818.s001.pdf]

# S1 file : Study Information Sheet

## Study Information Sheet (English version)

**(For the study participant)**

**Title : *Diagnostic Performance of Molecular and Serological Tests of SARS- CoV-2 on well-Characterised Specimens from COVID-19 Individuals : the "PERFECT-Study"* (PERFormance Evaluation of COVID-19 Tests)**

**Acronym**: PERFECT (PERFormance Evaluation of COVID-19 Tests)

**Principal investigator** : Dr Fokam Joseph

**<u>Invitation for your participation into a study on COVID-19 testing</u>**

**Research objectives** : To evaluate the diagnostic performance of SARS-COV2 using real-time PCR, rapide antigen and serological assays for sero-surveillance.

**Target population** : Participants (aged 21 years and above) tested for SARS-CoV-2.

**Study period** : The study will run for tweenty four months, as from ethical approval.

**Study procedure** : This is an observational and cross-sectional study during which you will be provided with an information notice on the present study ; detailed explanation on the study with be done for your perusal, and then a written informed consent will be provided prior to enrollment. If you are eligible, nasopharyngeal/oropharyngeal swabs and blood/serum specimens will be collected by trained and experienced personnels. Swabs will be stored in the viral transport media (for real time PCR) while blood/serum will be stored in test tubes (for serology/rapid testing respectively).

**Study participation** : You are free to accept participating into the study, and your participation will remain voluntary throughout the project ; you therefore have full right to refuse and withdraw your consent at anytime, without any coercion or without any implication on your case management. A copy of your signed informed consent will be at your personal disposal.

**Confidentiality** : Privacy and confidentiality will be ensured, through the use of unique identifiers and an encrypted database, to protect personal information of participants. Participants will be free to deliberately leave the study at any time, without any effect.

**Benefits and risk management** : nasopharyngeal swab collection is slightly disagreeable but non-invasive and will be done by qualified staffs. Some minor psychological risks may occur while filling the questionnaire. RT-PCR results will be issued free of charge for clinical diagnosis of COVID-19. Findings will be communicated for improved diagnosis, management and surveillance of COVID-19.

**For further details, you can contact the principal investigator**: Dr Joseph Fokam ; Head of the Virology

Laboratory of the CIRCB – Chantal BIYA Internaional Rference Centre for research on HIV/AIDS preventioin and management, Melen, Yaoundé; téléphone: 222235450; email: fokamjoseph@circb.cm, josephfokam@gmail.com

**Address of the National Ethics Committee for Research on Human Health that issued ethical clearance for the study**: telephone: 243674339; email: setcominae@gmail.com.

# Notice d'information de l'étude (French version)

**(Pour le participant de l'étude)**

**Titre :** ***Evaluation de la Performance des Outils de Diagnostic Moléculaire et Sérologique du SRAS-CoV-2 sur des Echantillons bien caractérisés des Individus Testés pour la COVID-19 : Etude "PERFECT" (PERFormance Evaluation of COVID-19 Tests)***

**Investigateur Principal** : Dr Joseph Fokam

**<u>Invitation pour la participation de votre enfant à l'étude sur le diagnostic de la COVID-19</u>**

**Objectifs de l'étude :** Evaluer la performance diagnostique du SRAS-COV2 des tests de PCR en temps reel, des tests rapides antigeniques pour le diagnostic et des tests serologiques pour la sero-surveillance.

**Population cible** : Participants (agés de 21 ans et plus) testés pour l'infection au SRAS-CoV-2.

**Période d'étude** : La durée est de 24 mois, dès disponibilité de la clairance ethique.

**Déroulement de l'étude** : Il s'agit d'une etude observationnelle et transversale, durant laquelle une notice d'information sur la présente étude ; des explications détaillées sur l'étude vous seront données au besoin ; et puis un consentement éclairé sera signé avant enrolement. En cas d'eligibilité, les échantillons nasopharyngés/oropharyngés et de sang/sérum seront collectés par des personnels experiemntés et formés. Les écouvillons de prelevement seront conservés dans des materiels de transport viral (pour le test de PCR), alors que le sang/serum sera conservé (pour les tests serologiques respectifs).
Le matériel biologique résiduel sera conservé pendant cinq ans au maximum, pour d'éventuelles investigations approfondies, et tout résultat probant sera communiqué au participant pour sa prise en charge bioclinique et son bien être personnel. Après la période de conservation, tout échantillon biologique encore disponible sera détruit.

**Participation à l'étude** : Vous êtes entièrement libre d'autoriser la participation de votre enfant à l'étude, et cette participation sera toujours volontaire tout au long de l'étude ; vous ainsi le plein droit de refuser ou de retirer votre consentement à tout moment, sans contrainte ni aucune implication sur la prise en charge de votre enfant. Une copie signée de votre consentement éclairé vous sera remise pour votre propre gouverne.

**Nécessités, bénéfices et risques liés à l'étude** : Le prelevement nasopharyngé est legerement désagréable et non-invasif, et sera réalisé par un personnel formé et qualifié. Quelques risques psychologiques mineurs pourraient se ressentir lors du remplissage de la fiche de questionnaire, mais ceux-ci sont tolerables et sans effet considerable. Comme bénéfice direct, les examens d'analyse seront pris en charge et les résultats, y compris les tests d'anticorps, d'antigene, de PCR et de sequencage du SARS-CoV-

2 en cas d'infection, et ces resultats seront rendus gratuitement pour toute benefice clinique individuel du participant et aussi pour un lien à la prise en charge en cas de besoin.

**Pour tout détail, contacter l'investigateur principal**: Dr Joseph Fokam, Virologue; chef du Laboratoire de Virologie du Centre International de Référence Chantal BIYA pour la recherche sur la prévention et la prise en charge du VIH/SIDA) à Melen, Yaoundé; téléphone: 222235450; email: fokamjoseph@circb.cm, josephfokam@gmail.com

**Adresse du Comité National d'Ethique pour la Recherche en Santé Humaine, ayant délivre la clairance éthique pour l'étude**: téléphone: 243674339; email: : setcominae@gmail.com.
