# Supplementary material for: Diagnostic performance of molecular and serological tests of SARS-CoV-2 on well-characterised specimens from COVID-19 individuals: The EDCTP "PERFECT-study" protocol (RIA2020EF-3000)
Source: PLoS One. 2022 Sep 21;17(9):e0273818. doi: 10.1371/journal.pone.0273818 (PMC9491536; doi:10.1371/journal.pone.0273818)
Supplement: S2 File — (PDF) [file pone.0273818.s002.pdf]

S2 file : Informed consent

CONSENT FORM (English version)

INFORMED CONSENT

(For the study participant)

I the undersigned, Mr/Mme/Mlle [Name(s) and surname(s)]:…………………………………………., legal guardian of [Name(s) and surname(s)]:……………………………………………………………

Acknowledge to have been invited to provide an informed consent for the study entitled: "***Diagnostic Performance of Molecular and Serological Tests of SARS- CoV-2 on well-Characterised Specimens from COVID-19 Individuals: the "PERFECT-Study" (PERFormance Evaluation of COVID-19 Tests)***", of which the Principal Investigator is named Dr Joseph Fokam, Medical Virologist and head of the Virology Laboratory at the Chantal BIYA International Reference Centre for research on HIV/AIDS prevention and management), Melen, Yaounde; telephone: 222235450; email: fokamjoseph@circb.cm, josephfokam@gmail.com.

- I have read and well understood the study information sheet provided;
- Or the study information sheet has been read and explained to me;
- I have well understood the goals and objectives of the study;
- I have received all relevant answers to my questions regarding the study;
- The study requirements, risks and benefits have been presented and explained;
- I well understood that I am deliberately free to accept or refuse to participate;
- My consent does not discharge the study investigators from their responsibilities;
- I preserve all my rights as per legal regulations.

Thus, I freely and voluntarily accept to participate within the conditions mentioned in the study information sheet, namely:

- ✓ To provide medical information necessary within the frame of the study;
- ✓ To watch and inform on any relevant health issue within study scope;
- ✓ To ensure attendance to any planed appointments for the study;
- ✓ To allow the collection of 6ml of whole blood and naso/oropharyngeal swabs for laboratory analyses;
- ✓ To ensure the appropriate use of laboratory results provided within the study;
- ✓ To authorise the storage of residual samples within the study for subsequent investigations, and to receive any relevant results thereof.

I therefore provide acceptance for my participation to this study.

Place: ……………………………., on the……/……/………

Study Investigator	The participant
(Name and address)	(Name and address)
Signature	Signature

FORMULAIRE DE CONSENTEMENT (French version)

CONSENTEMENT ECLAIRE
(Pour le participant à l'étude)

Je soussigné, Mr/Mme/Mlle [Nom(s) et Prénom(s)] :……………………………………………………
Parent légal de [Nom(s) et Prénom(s)] :…………………………………………………………………
Reconnais avoir été invité à donner mon accord au travail de recherche intitulé «**Evaluation de la Performance des Outils de Diagnostic Moléculaire et Sérologique du SRAS-CoV-2 sur des Echantillons bien caractérisés des Individus Testés pour la COVID-19: Etude "PERFECT"** ***(PERFormance Evaluation of COVID-19 Tests)***», dont l'investigateur Principal s'appelle Dr Joseph Fokam, Virologue et chef du Laboratoire de Virologie au Centre International de Référence Chantal BIYA pour la recherche sur la prévention et la prise en charge du VIH/SIDA) à Melen, Yaoundé; téléphone: 222235450; email: fokamjoseph@circb.cm, josephfokam@gmail.com.

- J'ai lu et bien compris la notice d'information qui m'a été remise concernant l'étude ;
- Ou bien on m'a lu et expliqué la notice d'information relative à cette étude ;
- J'ai bien compris l'intérêt et les objectifs de cette étude ;
- J'ai reçu toutes les réponses aux questions que j'ai posées ;
- Les nécessités, risques et bénéfices liés à l'étude m'ont été présentés et expliqués ;
- J'ai bien compris que je suis libre d'accepter ou de refuser d'y participer ;
- Mon consentement ne décharge pas les investigateurs de la recherche de leurs responsabilités ;
- Je conserve tous mes droits tel que garantis par la loi.

Ainsi, j'accepte librement de participer dans les conditions précisées dans la notice d'information de l'étude, notamment :

- ✓ De fournir les informations médicales nécessaires durant l'étude ;
- ✓ De veiller à la notification précise de mon état de santé en rapport à l'étude ;
- ✓ De veiller aux respects de tout rendez-vous programmé dans le cadre de l'étude ;
- ✓ D'autoriser le prélèvement de 6ml de sang total et des prelevements naso/oropharyngés pour les analyses de laboratoire liées à l'étude ;
- ✓ De veiller au bon usage des résultats fournis dans le cadre de l'étude ;
- ✓ D'accorder que le reste des échantillons prélevés dans le cadre de cette étude soit conservé pour des études ultérieures, et que tout resultat pertinent me soit communiqué à toute fin utile.

Je donne donc mon accord pour ma participation à cette étude.

Fait à……………………………, le……/……/………

Investigateur Principal du site d'étude (Nom et adresse)	Participant (Nom et adresse)
Signature	Signature
