# Supplementary material for: Diagnostic performance of molecular and serological tests of SARS-CoV-2 on well-characterised specimens from COVID-19 individuals: The EDCTP "PERFECT-study" protocol (RIA2020EF-3000)
Source: PLoS One. 2022 Sep 21;17(9):e0273818. doi: 10.1371/journal.pone.0273818 (PMC9491536; doi:10.1371/journal.pone.0273818)
Supplement: S3 File — (PDF) [file pone.0273818.s003.pdf]

S3 file : Data collection tool

Project title: "Diagnostic Performance of Molecular and Serological Tests of SARS- CoV-2 on well-Characterised Specimens from COVID-19 Individuals: the "PERFECT-Study"

REPUBLIQUE DU CAMEROUN<br>Paix – Travail – Patrie<br>------------------<br><br>MINISTERE DE LA SANTE PUBLIQUE<br>------------------	REPUBLIC OF CAMEROON<br>Peace – Work – Fatherland<br>--------------------<br><br>MINISTRY OF PUBLIC HEALTH<br>--------------------

FORMULAIRE DE NOTIFICATION ET D'INVESTIGATION DES CAS DE « COVID-19 » AU CAMEROUN

**EPI ID :** CO-CMR-/__/__/__/-/__/__/__/- /__/__/__/__/__/__/<br>*Code Région (3) Code district (3) Numéro d'ordre (5)*	**Date de remplissage :** ____/____/20___/
**Région :** _________ **District :** ___________________ **Aire de santé :** __________________ Lieu : _________________________	

1. Données socio-démographiques

Statut du cas : ☐ Vivant ☐ Décédé si décédé, date de décès /______/________/20______/

Nom et prénom du cas : ____

Sexe : ☐ M ☐ F **Âge :** ____ ☐ Années ☐ Mois **Date de naissance :** /______/_______/________/

Pays de résidence : _______________ Ville : _________________ **Nationalité :** ______________ **Tél. :** ________________

N CNI ou Passeport : ________________ **Profession :** _______________________ **Professionnel de santé :** ☐Oui ☐Non

2. Signes et symptômes

☐ Asymptomatique Date de début des symptômes /_______/________/20_______/

Fièvre (t°≥38 °C)	☐ Oui ☐ Non ☐ NSP	Éruption cutanée	☐ Oui ☐ Non ☐ NSP
Frissons	☐ Oui ☐ Non ☐ NSP	Conjonctivite	☐ Oui ☐ Non ☐ NSP
Toux	☐ Oui ☐ Non ☐ NSP	Essoufflement	☐ Oui ☐ Non ☐ NSP
Mal de gorge	☐ Oui ☐ Non ☐ NSP	Douleurs musculaires	☐ Oui ☐ Non ☐ NSP
Écoulement nasal	☐ Oui ☐ Non ☐ NSP	Difficultés à respirer	☐ Oui ☐ Non ☐ NSP
Vomissements	☐ Oui ☐ Non ☐ NSP	Céphalées	☐ Oui ☐ Non ☐ NSP
Diarrhée	☐ Oui ☐ Non ☐ NSP	Perte de saveur	☐ Oui ☐ Non ☐ NSP
Perte de l'odorat	☐ Oui ☐ Non ☐ NSP	Fatigue intense	☐ Oui ☐ Non ☐ NSP

Autres, préciser : ____

3. Comorbidités

**Cancer**	☐Oui ☐Non ☐ NSP	**VIH**	☐Oui ☐Non ☐ NSP
**Diabète**	☐Oui ☐Non ☐ NSP	**Maladie cardiaque**	☐Oui ☐Non ☐ NSP
**HTA**	☐Oui ☐Non ☐ NSP	**Insuffisance rénale**	☐Oui ☐Non ☐ NSP
**Obésité**	☐Oui ☐Non ☐ NSP	**Drépanocytaire**	☐Oui ☐Non ☐ NSP

Maladie pulmonaire chronique ☐Oui ☐Non ☐ NSP

Une aménorrhée/grossesse (uniquement sexe féminin). ☐Oui ☐Non ☐ NSP Si oui préciser le trimestre : ________

☐ **Autres préciser** : ______________________________

4. Antécédents

Avez-vous reçu le vaccin contre la COVID-19 ? ☐ Oui ☐ Non Si oui date de vaccination : _______/________/20_______
Nom du vaccin reçu :__________________________________ Nombre de doses reçu :________

Avez-vous déjà été diagnostiqué positif COVID-19 ? ☐Oui ☐Non, Si oui avez-vous été déclaré guéri ? ☐Oui ☐Non

Avez-vous voyagé durant les 14 derniers jours ? ☐Oui ☐Non . Si oui, préciser les lieux (villes, quartiers ?) :
____

5. Examen

Nature du test : ☐TDR Ag ☐ TDR Ac ☐ PCR

Type de prélèvement : ☐Nasopharyngé ☐Autres **Date de prélèvement :** __________/____________/____________

Indication du prélèvement : ☐Volontaire ☐Contrôle ☐Contact ☐Autres

Résultats : ☐Présence de SARS Cov-Ag ☐Absence SARS Cov-Ag ☐Indéterminé

Conclusion : ☐Positif ☐Négatif ☐Indéterminé **Transmis pour séquençage génomique** : ? ☐ Oui ☐ Non

Manipulateur :____

Téléphone du manipulateur : ____

Noms de(s) l'investigateur(s) : ____

Numéro de téléphone : ____

Adresses email : ____

Antibody response	IgG/IgM positive	IgG positive or IgM positive	IgG/IgM negative
Antibody titer	BAU/ml	BAU/ml	BAU/ml
PCR :	Presence viral RNA	Absence viral RNA	Date : / /
If positive, viral strain		Date of sequencing :	Date : / /
